# Pressure induced superconductivity bordering a charge-density-wave state in NbTe_4_ with strong spin-orbit coupling

**DOI:** 10.1038/s41598-018-24572-z

**Published:** 2018-04-19

**Authors:** Xiaojun Yang, Yonghui Zhou, Mengmeng Wang, Hua Bai, Xuliang Chen, Chao An, Ying Zhou, Qian Chen, Yupeng Li, Zhen Wang, Jian Chen, Chao Cao, Yuke Li, Yi Zhou, Zhaorong Yang, Zhu-An Xu

**Affiliations:** 1 State Key Laboratory of Silicon Materials and Department of Physics, Zhejiang University, Hangzhou, 310027 China; 2 School of Physics and Optoelectronics, Xiangtan University, Xiangtan, 411105 China; 3 Anhui Province Key Laboratory of Condensed Matter Physics at Extreme Conditions, High Magnetic Field Laboratory, Chinese Academy of Science, Hefei, 230031 China; 4 Department of Physics, Hangzhou Normal University, Hangzhou, 310036 China; 5 Zhejiang California International NanoSystems Institute, Zhejiang University, Hangzhou, 310058 China; 6 Collaborative Innovation Centre of Advanced Microstructures, Nanjing, 210093 P. R. China

## Abstract

Transition-metal chalcogenides host various phases of matter, such as charge-density wave (CDW), superconductors, and topological insulators or semimetals. Superconductivity and its competition with CDW in low-dimensional compounds have attracted much interest and stimulated considerable research. Here we report pressure induced superconductivity in a strong spin-orbit (SO) coupled quasi-one-dimensional (1D) transition-metal chalcogenide NbTe_4_, which is a CDW material under ambient pressure. With increasing pressure, the CDW transition temperature is gradually suppressed, and superconducting transition, which is fingerprinted by a steep resistivity drop, emerges at pressures above 12.4 GPa. Under pressure *p* = 69 GPa, zero resistance is detected with a transition temperature *T*_*c*_ = 2.2 K and an upper critical field *μ*_0_*H*_*c*2_ = 2 T. We also find large magnetoresistance (MR) up to 102% at low temperatures, which is a distinct feature differentiating NbTe_4_ from other conventional CDW materials.

## Introduction

Transition-metal chalcogenides possess rich structural chemistry and a wide variety of unusual physical properties^1–3^. The latter includes, for instance, charge density wave (CDW)^3^, superconductivity^4–7^ and recently reported extremely large magnetoresistance in WTe_2_^8–13^. Among chalcogenides, tellurides are usually different from sulfides and selenides in crystal structures, electronic structures and physical properties, due to the diffusive nature of the tellurium valence orbitals^14^ and thus more covalent character of tellurium^1^. While sulfides and selenides, such as NbS_2_, NbSe_2_ and NbSe_3_, were intensively studied in the context of CDW and/or superconductivity^3,4,15^, tellurides have not received much attention until recently^8–13^. One import feature of tellurides is that the atomic number of Te is very large, resulting in a strong spin-orbital (SO) coupling. Nowadays, topological materials with strong SO coupling have been drawing plenty of attention in condensed matter physics^16^. It is highly desirable to discover noval superconductors with strong SO coupling for understanding the nature of topological superconductivity. On the other hand, low dimensionality is accompanied by strong lattice instability. Additional interest for pursuing a quasi-1D material with itinerant electrons comes from the possible realization of Luttinger liquid, in which an exotic spin-charge separation is expected^17^. Thus, it should be of great interest to study superconductivity in quasi-1D tellurides with large atomic number^14,18^, where competing interactions might give rise to interacting ground states.

The magnetoresistance (MR) in ordinary non-magnetic metals is a relatively weak effect and usually at the level of a few percent^19,20^. Materials exhibiting large MR are not only utilized to enlarge the sensitivity of read/write heads of magnetic storage devices, e.g., magnetic memory^21^ and hard drives^22^, but also stimulating many fundamental researches^8,23^. Typically, large negative MR occurs in thin-film metals^24^, manganese based perovskites^25,26^ and some disordered systems^27,28^, while large positive MR has been observed in semiconductors^29^ and semimetals^8,30^. In general, there exists only a few of CDW materials, which show large positive MR^31–33^. The origin of the huge positive MR effect in the CDW state is still under debate. Exploring more CDW materials with large MR may help to clarify the controversial explanations.

**Figure 1:**
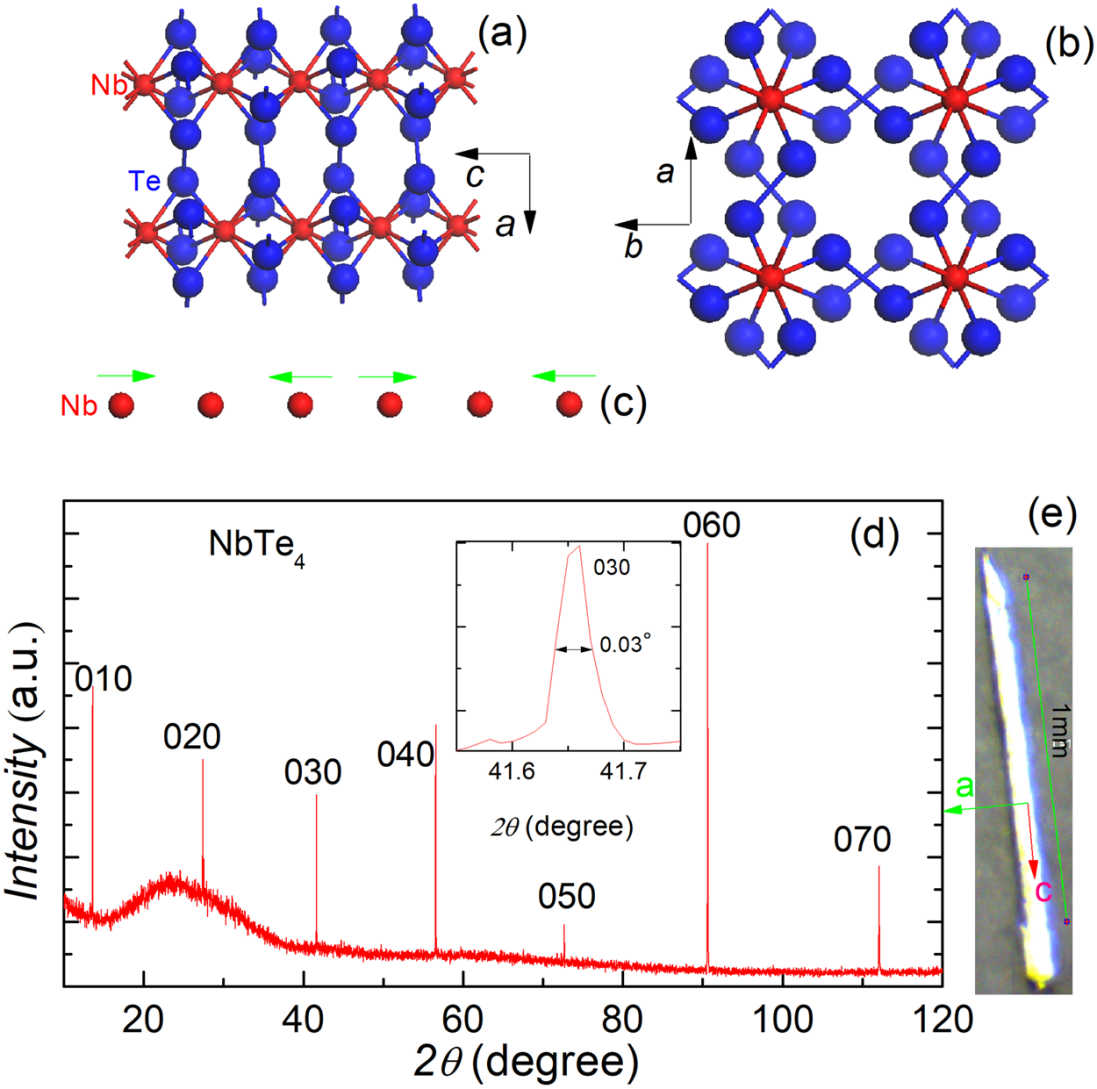
Structural characterization by x-ray diffractions for NbTe_4_. (**a**,**b**) The basic crystal structure of NbTe_4_ projected along the [010] **(a)** and [001] **(b)** directions. **(c)** The red solid circles represent equidistant Nb ions on the axis of a single column of the basic structure; the arrows show the displacements and resultant trimerizations of the Nb ions in the commensurate phase. **(d)** XRD structure characterization of a NbTe_4_ single crystal. Only (0 *l* 0) peaks can be observed. The inset: The enlarged XRD curve of (030) peak. **(e)** A typical crystal of NbTe_4_, with crystallographic directions marked.

NbTe_4_ and TaTe_4_ belong to the same group of quasi-1D CDW materials (space group P4/mcc). The structure of NbTe_4_ was first determined by Selte and Kjekshus in 1964^34^. The metal atoms Nb form linear chains along the tetragonal *c*-axis and the Te atoms form square antiprismatic formulae in which the metal atoms confined (Fig. 1(a,b)). Superlattice reflections indicate that the *a*-axis is doubled and the *c*-axis is tripled, leading to an enlarged unit cell 2a* × 2a* × 3c* ^34,35^. NbTe_4_ undergoes a strong lattice distortion around room temperature to form an incommensurate charge density wave (CDW) phase^35–37^. The resulting CDW superstructure in NbTe_4_ was visualized by scanning tunneling microscopy (STM)^37^. Intriguingly, the CDW ordering in NbTe_4_ is highly anisotropic due to the quasi-1D chain structure. Strikingly, there exist three CDW orders in NbTe_4_, two are incommensurate with wave vectors ${\overrightarrow{q}}_{1}$ = (0, 0, 0.311c*) and ${\overrightarrow{q}}_{2}$ = (0.5a*, 0.5b*, 0.344c*), respectively, and the third is commensurate with ${\overrightarrow{q}}_{3}$ = (0.5a*, 0, $\frac{1}{3}$c*)^35^. The crystal structure of NbTe_4_ is depicted in Fig. 1(a,b). The displacements of Nb ion in a single column for the commensurate phase are shown in Fig. 1c, which can also be found in previous literature^38^. Indeed, NbTe_4_ and TaTe_4_ are the only two reported crystals in which three CDWs coexist^35^. In this paper, we report large magneto-resistance and high pressure induced superconductivity in NbTe_4_. The CDW transition temperature is strongly suppressed by applied pressure, and superconductivity fingerprint of steep resistivity drop emerges when pressure exceeds 12.4 GPa. Under pressure *p* = 69 GPa, zero resistance is reached with a transition temperature *T*_*c*_ = 2.2 K and an upper critical field of 2 T. We also observed large magnetoresistance (MR) up to 102% at low temperatures in NbTe_4_, which is rarely observed in conventional CDW systems.

## Results and Discussions

Figure 1d displays the X-ray diffraction pattern of NbTe_4_ single crystal. Only multiple reflections of the (0 *l* 0) planes can be detected, consistent with the quasi-one-dimensional crystal structure depicted in Fig. 1(a,b). The interplane spacing is determined to be 6.499 ${\rm{\AA }}$, agreeing with the previous reported value of the NbTe_4_ phase^34^. For the (030) peak, the full width at half-maximum is only 0.03°, indicating high quality of the crystals.

**Figure 2:**
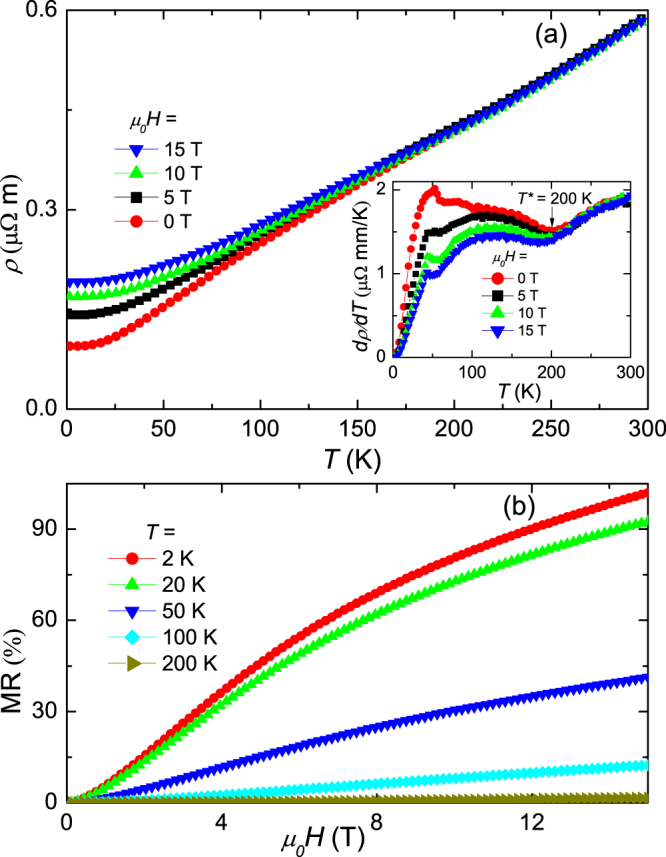
Transport properties of NbTe_4_ at ambient pressure. (**a**) Plots of resistivity against temperature under *μ*_0_*H* = 0, 5, 10, and 15 T. The current is along the *c*-axis, and the field is along the *b*-axis. Inset: Plots of d*ρ*/d*T* versus temperature. (**b**) Field dependence of ${\rm{MR}}=[\rho (H)-\rho \mathrm{(0)]/}\rho \mathrm{(0)}$ under various temperatures.

The temperature dependence of resistivity under various applied magnetic fields (*μ*_0_*H* up to 15 T) is summarized in Fig. 2a. In zero field, the room temperature resistivity is 59.2 $\mu {\rm{\Omega }}\cdot {\rm{cm}}$ and decreases to 9.4 $\mu {\rm{\Omega }}\cdot {\rm{cm}}$ at *T* = 2 K, yielding a residual resistivity ratio (RRR) of 6.3. Low RRR in NbTe_4_ single crystals has been commonly reported, which is irrespective of growth conditions^39,40^. To understand the origin of the poor RRR, we performed the measurements of energy dispersive X-ray spectroscopy (EDXS). The chemical composition determined by EDXS gives the atomic ratio Nb:Te = 0.8:4, with a measurement error of ±3.5% depending on the elements measured. This result indicates that there exists significant deficiency for Nb, which may be responsible for the poor RRR. To get further insight into the resistivity data, we take the partial derivative of resistivity with respect to temperature, as shown in the inset of Fig. 2a. At T^*^ = 200 K a cusp is observed, consistent with the previous reports^39,40^. It is known that the two incommensurate superlattices with ${\overrightarrow{q}}_{1}$ = (0, 0,0.311c*) and ${\overrightarrow{q}}_{2}$ = (0.5a*, 0.5b*, 0.344c*) are stable at room temperature in NbTe_4_. Below room temperature, additional superlattice ordering with ${\overrightarrow{q}}_{3}$ = (0.5a*, 0, $\frac{1}{3}$c*) ermerges at about 200 K. The change of the slope in the temperature dependence of resistivity at 200 K should be due to the appearance of the ${\overrightarrow{q}}_{3}$ superlattice^39,40^. Usually, more drastic anomalies in resistivity should be observed at the CDW transition temperatures. For example, two sharp increases in resistivity were observed in NbSe_3_ at 145 and 59 K^15^, corresponding to about 20% and 48% of conduction electrons condensating into the CDW states, respectively^15,33^. The observed small change of resistivity around 200 K in NbTe_4_ is indicative of a finite but slight decrease of free carrier density associated with the reconstruction of the Fermi surface by Brillouin zone refolding. The anomaly at around *T*_*L*_ = 50 K may be due to the lock-in transition into the 2a* × 2a* × 3c* superstructure, which is worthy of further clarification^40^. The anomies at T^*^ = 200 K and *T*_*L*_ = 50 K, which were also observed in previous reports^39,40^, further confirm the CDW features observed in NbTe_4_.

The field (*H*//*b*) dependence of resistance (*I*//*c*) at various temperatures is shown in Fig. 2b. The magneto-resistance (MR), which is defined as ${\rm{MR}}=[\rho \mathrm{(15}T)-\rho \mathrm{(0)]/}\rho \mathrm{(0)}$, rises up to 102% under a magnetic field of 15 T at *T* = 2 K. Although the MR value of NbTe_4_ is orders of magnitude smaller than that of WTe_2_^8^ and Bi^30^, it is much larger than that of a usual single-band weakly interacting electron system, in which the MR is usually at the level of a few percent^8,20^. For a single-band noninteracting electron system, the Hall field exactly balances the Lorentz force, and the electron moves as if in zero field without being deflected; thus, there is no remarkable magnetoresistance^20,41^.

**Figure 3:**
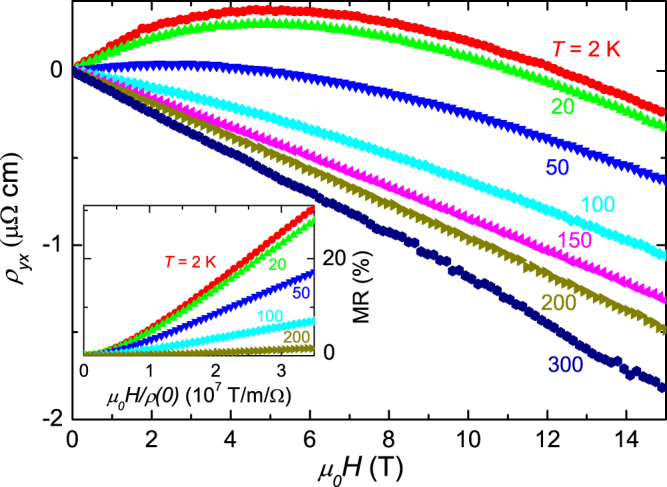
Hall resistivity and Kohler plot for NbTe_4_. Field dependence of Hall resistivity (*ρ*_*yx*_) under various temperatures. The current is along the *c*-axis, and the field is along the *b*-axis. The inset: Kohler’s rule by plotting the MR vs. ${\mu }_{0}H/\rho \mathrm{(0)}$ from 2 K to 200 K.

Figure 3 displays the field (*H*//*b*) dependence of Hall resistivity (*I*//*c*) *ρ*_*yx*_ at various temperatures. At 2 K, *ρ*_*yx*_ is positive under low fields but switches to negative sign in higher fields. With increasing temperature, the required field where *ρ*_*yx*_ changes its sign decreases. The curvature and sign reversal in the Hall resistivity indicate the coexistence of hole-type minority carriers with high mobility and electron-type majority carriers with low mobility^42,43^. The multiband nature of NbTe_4_ is also manifested in the breakdown of the Kohler’s rule, as plotted in the inset of Fig. 3. According to the Kohler’s rule, if only one relaxation time *τ* exists in metals, then MR can be characterized by a function of ${\mu }_{0}H/\rho \mathrm{(0)}$, and the results for different temperatures should collapse into a single curve^41,44^. As shown clearly in the inset of Fig. 3, the Kohler’s rule is violated in NbTe_4_, as evident by the non-overlapping of the MR curves at different temperatures. The observeation indicates more than one relaxation time *τ* exist, supporting the multiband result obtained by the Hall measurements.

Materials exhibiting a large magneto-resistance have potential device applications and thus have been attracting researchers’ interest^45^. The most well-known examples are the giant magnetoresistance in magnetic multilayers^24^ and colossal magnetoresistance in manganites^26^, both of them rely on the coupling between spin configuration and charge transport. However, even among nonmagnetic materials, extremely large magnetoresistance (MR) may arise in semimetals with electron-hole Fermi surface (FS) compensation^46–49^.

As a discussion of the multiband effects in NbTe_4_, we define the direction of the current (Hall voltage) as the *x*-axis (*y*-axis). Even though no net current should exist in the *y*-direction, the currents in the *y*-direction by one particular type of carriers may be non-zero in a multiband system. When we applied magnetic field, the *y*-direction currents should be affected by the Lorentz force which is antiparallel to the *x*-direction^50^. The back flow of carriers provides a substantial source of the large magnetoresistance in metals with multiple bands like MgB_2_^41^ and semimetals like Bi^48^ and WTe_2_^8^. Owing to the coexistence of both electron- and hole-type carriers, the large MR in NbTe_4_ may be attributed to multiband effects. We employed a two band mode to fit the *ρ*_*xx*_ and *ρ*_*yx*_ data simultaneously in the low field region (See supplementary material for a simultaneous fitting of *ρ*_*xx*_ and *ρ*_*xx*_ data by the two band semiclassical model). The values of *n*_*e*_ and *n*_*h*_ are close to each other at all the measured temperatures, which suggests that the large MR should result from the electron-hole compensation effect. The mobilities *μ*_*e*_ and *μ*_*h*_ increase with decreasing temperatures as the usual metals. Meanwhile, the carrier concentrations decrease significantly at low temperatures, which occurs commonly in a CDW system. At *T* = 2 K, *μ*_*e*_ is 0.22 m^2^V^−1^s^−1^, while *μ*_*h*_ is 0.29 m^2^V^−1^s^−1^. Actually large magnetoresistance was also observed in the CDW materials NbSe_3_^31,33^ and AMo_6_O_17_ (A = Na, K, and Tl)^32^. It has been proposed that the large MR in these CDW systems may result from the magnetic-field-induced enhancement of the CDW gap^31^, but a study by Tritt *et al*.^51^ presented negative results on such a claim. Until now, the nature of the huge positive MR effect in the CDW state is still ambiguous. Actually, only a few CDW materials show large positive MR^31–33^. The origin of the large MR in NbTe_4_ deserves further investigation.

Superconductivity often occurs in the proximity of other competing ordered states. Both high-*T*_*c*_ cuprates and Fe-based superconductors are close to antiferromagnetic (AFM) ordered states^52,53^. The AFM order in Fe-based superconductor is of a spin density wave (SDW)-type. For CDW materials, high pressure or chemical doping can continuously suppress the CDW order, and then superconducting transition temperature is enhanced or superconducting state emerges after the suppression of CDW state^54,55^. The weakening of competing orders normally favors superconductivity^56^. The CDW order of NbTe_4_ survives up to very high temperatures. Thus it is interesting to investigate whether superconductivity can be induced by pressure.

**Figure 4:**
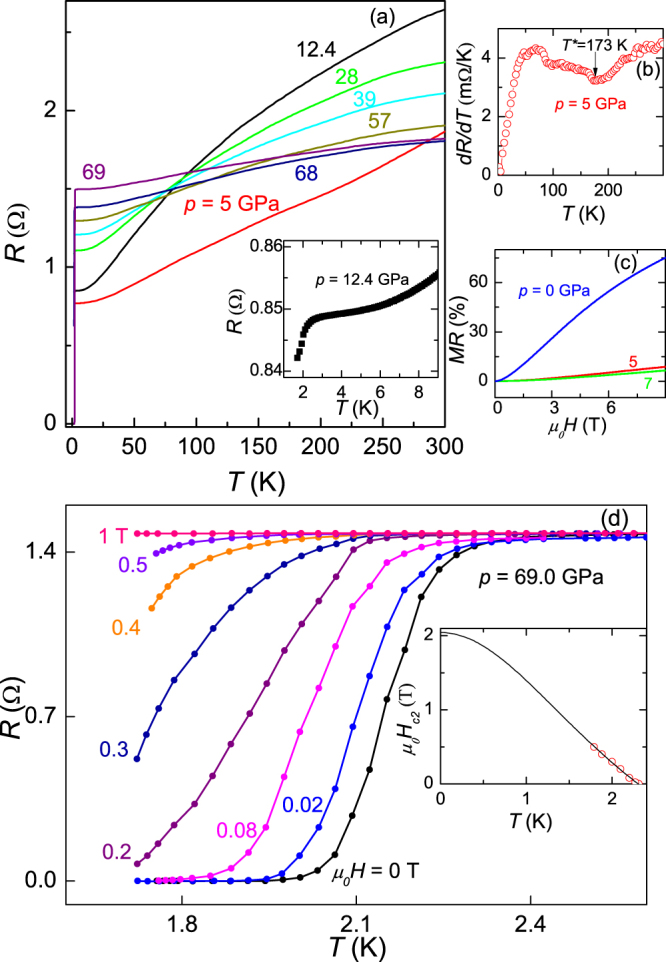
Transport properties for single crystal NbTe_4_ under pressure. **(a)** The plot of resistance versus temperature for the pressure ranging from 5 GPa to 69 GPa. The current is along the *c*-axis. The inset: The enlarged plot of resistance at low temperatures under *p* = 12.4 GPa. **(b)** Plots of d *R*/d *T* versus temperature under *p* = 5 GPa. **(c)** Field dependence of MR under *p* = 0, 5 and 7 GPa. **(d)** Temperature dependence of resistance for several different magnetic fields under *p* = 69 GPa. The inset displays the upper critical fields as a function of temperatures.

Figure 4a shows the evolution of resistance (*I*//*c*) as a function of temperature of the NbTe_4_ single crystal at various pressures from 5 GPa to 69 GPa. The samples used in high pressure and ambient pressure measurements are two different ones, but they are from the same batch. Under an applied pressure of *p* = 5 GPa, the cusp associated with the appearance of the ${\overrightarrow{q}}_{3}$-superlattice CDW transition is suppressed to T^*^ = 173 K, as shown in Fig. 4b. The MR decreases to less than 10% with an applied pressure of 5 GPa, and becomes smaller and smaller with an increasing pressure, as shown in Fig. 4c. When the pressure increases up to 12.4 GPa, the cusp associated with the CDW transition is suppressed and becomes too weak to be distinguished in the resistivity curve, instead, a sudden resistivity decrease presents at *T*~2.4 K, which could be a fingerprint of superconductivity. So we keep increasing the pressure to trace the superconducting transition. Upon further increasing pressure, at *T* = 1.7 K, which is the base temperature of our high pressure measurement system, the resistance drops to very small. Finally, under *p* = 69 GPa, which is the highest pressure we can apply, zero resistance has been detected. The low-T resistance appears to increase monotonically with pressure. In contrast, the room-temperature resistance keeps increasing up to 12.4 GPa, and then steadily decreases, which coincides with the disappearance of the CDW phase. Whether the variation in room-temperature resistance is correlated with the CDW order deserves further clarification. T_*c*_ around 2.2 K is stabilized for the pressure range from 10 to 69 GPa. Such a wide stabilization pressure range could be a result of heavy doping due to Nb deficiency up to 0.2. Figure 4d shows the suppression of superconductivity under magnetic fields (*H*//*b*). The inset gives the temperature dependence of the upper critical field ${\mu }_{0}{H}_{c2}(T)$, determined by using 90% normal state resistivity criterion. The temperature dependence of ${\mu }_{0}{H}_{c2}(T)$ is nearly linear in the investigated temperature range. According to the Ginzburg-Landau theory, the upper critical field *H*_*c*2_ evolves with temperature following the formula ${H}_{c2}(T)={H}_{c2}\mathrm{(0)}(1-{t}^{2})/(1+{t}^{2})$, where *t* is the renormalized temperature *T*/*T*_*c*_. It is found that the experimental upper critical field ${\mu }_{0}{H}_{c2}(T)$ can be well fitted by this model and its zero-temperature limit is extracted to be 2.0 T. Due to the limited temperature range, the estimated ${\mu }_{0}{H}_{c2}\mathrm{(0)}$ by this way may be of considerable error.

**Figure 5:**
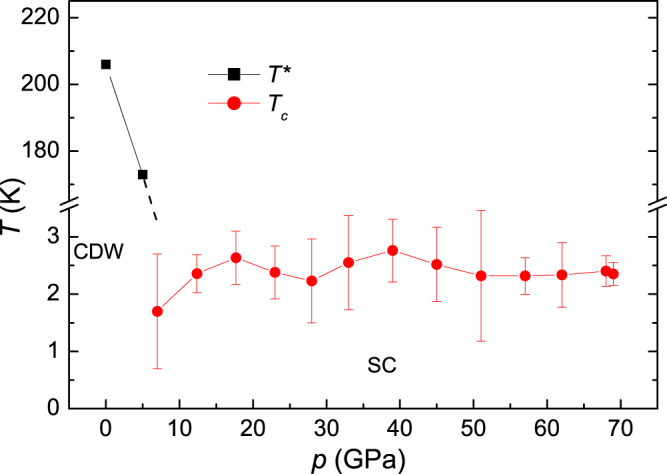
Temperature versus pressure phase diagram of NbTe_4_. T^*^ and *T*_*c*_ denote the resistivity anomaly temperature and the superconducting transition temperature, respectively.

The temperature-pressure phase diagram is summarized in Fig. 5. The CDW transition temperature in NbTe_4_ is strongly suppressed with an applied high pressure. Accompanying the suppression of the CDW order, superconductivity fingerprint appears. Such superconducting phase diagrams, where superconductivity may compete with different kind of orders, have been observed in many systems, including high-*T*_*c*_ cuprates, Fe-based superconductors, NbSe_2_ and Cu_*x*_TiSe_2_^52–55^. Barath *et al*. proposed that the superconducting paring mechanism in Cu_*x*_TiSe_2_ could be associated with the quantum criticality, stemming from the fluctuations of CDW order^57^. There are also many theoretical works on the superconducting dome in transition metal dichalcogenides (TMDCs), and pursuing the nature of the observed superconducting states is still on-going^58,59^. High temperature high pressure treatment could induce phase transformation in NbTe_4_^60^, which may be relevant to the occurrence of superconductivity. Further investigation on this issue is of potential interest. Since the physical properties of NbTe_4_ and TaTe_4_ show numerous similar features, the further research on pressure effect of TaTe_4_ should be gainful^61^. As a low dimensional chalcogenide, it could be fascinating to explore whether intercalation or doping could induce superconductivity, or at least decrease the critical superconducting pressure, as the case in Cu_*x*_TiSe_2_^57^, Cu_*x*_Bi_2_Se_3_^62^, Ir_1−*x*_ Pd_*x*_Te_2_^16^, (NbSe_4_)_3.33_I^63^. Primarily because of the strong SO coupling, the appealing Majorana surface state has been suggested to exist in Cu_*x*_Bi_2_Se_3_ superconductor^62,64^. Due to the large atom number in NbTe_4_, it is expected to own strong SO coupling. Furthermore, a recent theoretical work has proposed monolayer hole-doped TMDCs as candidates for topological superconductors^65^, which makes transition metal chalcogenide systems more fascinating. Thus it becomes quite interesting to explore the possible presence of nonconventional quantum states in NbTe_4_. Our work may provide another promising system for exploring novel topological superconductors.

## Summary

In conclusion, we discovered unusually large magnetoresistance(MR) and pressure induced superconductivity with a maximum *T*_*c*_ of 2.2 K in a quasi-one-dimensional (1D) transition-metal chalcogenide NbTe_4_ with strong spin-orbit coupling. Superconductivity appears when the CDW order is suppressed by high pressure. Although *T*_*c*_ is relatively low, the large MR and strong SO coupling make NbTe_4_ a promising candidate for the exploration of novel superconductors.

## Methods

### Sample synthesis and characterization

The NbTe_4_ crystals were grown by a self-flux method. Powders of the elements Nb (99.97%) and Te (99.99%), all from Alfa Aesar, in an atomic ratio of Nb:Te = 1:8 were thoroughly mixed together, loaded, and sealed into an evacuated quartz ampule. The ampule was slowly heated up to 1273 K and held for 25 h. After that, it was slowly cooled to 873 K at a rate of 3 K/h, followed by furnace cooling down to room temperature. Shiny, gray-black soft crystals in flattened needle shapes were harvested with a typical dimension of 1.2 × 0.02 × 0.02 mm^3^, as shown in Fig. 1(e). Single crystal X-ray diffraction (XRD) was performed at room temperature using a PANalytical X-ray diffractometer (Model EMPYREAN) with a monochromatic CuK_*α*1_ radiation. Energy-dispersive x-ray spectroscopy (EDXS) was collected by an Octane Plus Detector (AMETEX EDAX), equipped in a field-emitting scanning electron microscope (SEM, Hitachi S-4800).

### Measurements

The resistance data was collected using standard four-probe method in a screw-pressure-type diamond anvil cell (DAC), which is made of non-magnetic Cu-Be alloy. The diamond culet was about 300 *μ*m in diameter. A T301 stainless steel gasket was pre-indented from a thickness of 250 *μ*m to 35 *μ*m, leaving a pit inside the gasket. A hole with diameter of 300 *μ*m was drilled in the center of the pit by laser ablation. The pit of the indented gasket was then covered with a mixture of epoxy and fine cubic boron nitride (c-BN) powder and compressed tightly to insulate the electrode leads from the metallic gasket. Next, the c-BN-covered pit served as the sample chamber, where a NbTe_4_ single crystal in dimension of 200 *μ*m × 35 *μ*m × 5 *μ*m was inserted without the pressure-transmitting medium, together with a ruby ball served as a pressure marker at the top of the sample. The value of the pressure was determined by the ruby fluorescence method. Platinum (Pt) foil with a thickness of 5 *μ*m was used as electrodes. The gasket surface outside the pit was insulated from the electrode leads by a layer of Scotch tape. The DAC was put inside a home-made multifunctional measurement system (1.8–300 K, JANIS Research Company Inc.; 0–9 T, Cryomagnetics Inc.) with helium (He) as the medium for heat conduction to obtain high efficiency of heat transfer and good precision of temperature control. Two Cernox resistors (CX-1050-CU-HT-1.4 L) located near the DAC were employed to ensure the accuracy of temperature in the presence of magnetic field. The ambient-pressure electrical transport measurements were carried out in a Oxford cryostat system with magnetic field up to 15 T and temperature down to 1.5 K. Ohmic contacts were made with gold wires and silver paste.

## Electronic supplementary material

Supporting Materials
