# Supplementary material for: Pressure induced superconductivity bordering a charge-density-wave state in NbTe4 with strong spin-orbit coupling
Source: Sci Rep. 2018 Apr 19;8:6298. doi: 10.1038/s41598-018-24572-z (PMC5908920; doi:10.1038/s41598-018-24572-z)
Supplement: Supplementary file 1 — Supporting Materials [file 41598_2018_24572_MOESM1_ESM.pdf]

Supporting Materials for "Pressure induced superconductivity bordering a charge-density-wave state in $NbTe_4$ with strong spin-orbit coupling"

Xiaojun Yang[1,2], Yonghui Zhou[3], Mengmeng Wang[1], Hua Bai[1], Xuliang Chen[3], Chao An[3], Ying Zhou[3], Qian Chen[1], Yupeng Li[1], Zhen Wang[1], Jian Chen[1], Chao Cao[4], Yuke Li[4], Yi Zhou[1,6], Zhaorong Yang[3*] and Zhu-An Xu[1,5,6†]

[1] *State Key Laboratory of Silicon Materials and Department of Physics, Zhejiang University, Hangzhou 310027, China.*

[2] *School of Physics and Optoelectronics, Xiangtan University, Xiangtan 411105, China*

[3] *Anhui Province Key Laboratory of Condensed Matter Physics at Extreme Conditions, High Magnetic Field Laboratory, Chinese Academy of Science, Hefei 230031, China*

[4] *Department of Physics, Hangzhou Normal University, Hangzhou 310036, China*

[5] *Zhejiang California International NanoSystems Institute, Zhejiang University, Hangzhou 310058, China*

[6] *Collaborative Innovation Centre of Advanced Microstructures, Nanjing 210093, P. R. China*

(Dated: April 11, 2018)

* Electronic address: zryang@issp.ac.cn

† Electronic address: zhuan@zju.edu.cn

The field dependences of ρ_{xx} and ρ_{yx} are fit by the following equations:

$$\rho_{xx}(B) = \frac{1}{e}\frac{(n_h\mu_h + n_e\mu_e) + (n_h\mu_e + n_e\mu_h)\mu_h\mu_e B^2}{(n_h\mu_h + n_e\mu_e)^2 + (n_h - n_e)^2\mu_h^2\mu_e^2 B^2} \quad (1)$$

$$\rho_{yx}(B) = \frac{B}{e}\frac{(n_h\mu_h^2 - n_e\mu_e^2) + (n_h - n_e)\mu_h^2\mu_e^2 B^2}{(n_h\mu_h + n_e\mu_e)^2 + (n_h - n_e)^2\mu_h^2\mu_e^2 B^2} \quad (2)$$

where n_e, n_h, μ_e and μ_h are the carrier densities and mobilities of electrons and holes, respectively. Simultaneously fitting our ambient pressure data with the above equations in the low field region ($B \leq 1$ T), we can get the temperature dependences of n_e, n_h, μ_e and μ_h. The fitted curves are displayed as lines in Fig. S1(a) and S1(b) and they agree well with the experimental data in the field region of 0 T - 1 T.

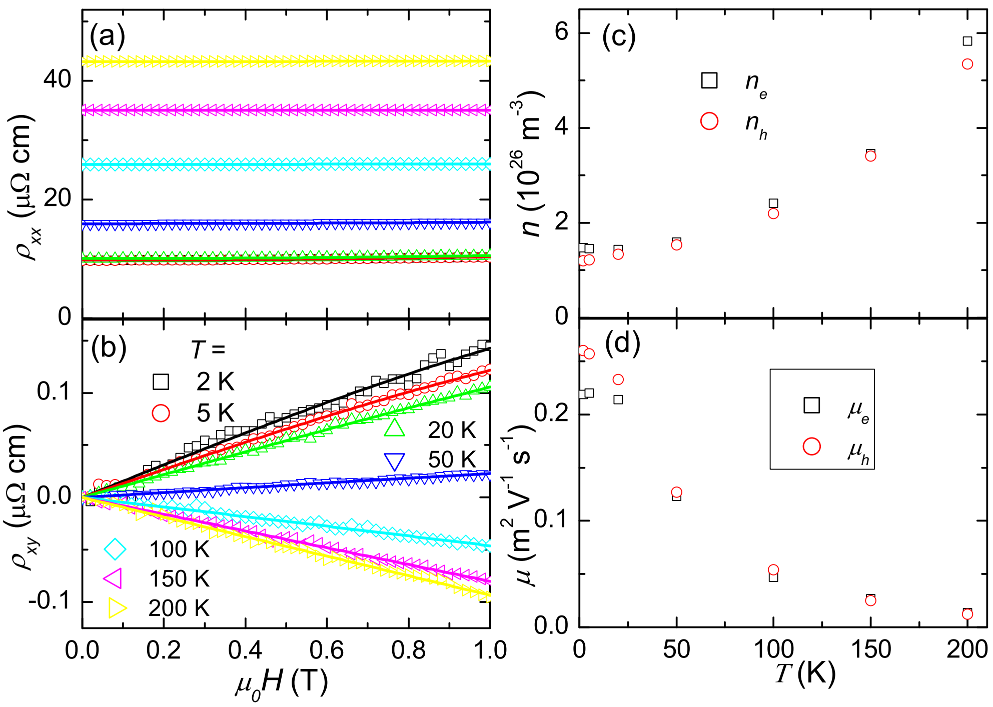

FIG. S 1: **Two band model fitting of low field transport properties of $NbTe_4$ single crystal with I//c-axis and B//b-axis.** Field dependence of **(a)**, transverse magnetoresistivity ρ_{xx} and **(b)**, Hall resistivity ρ_{yx}. The symbols correspond to experimental data, while the lines are the fitting curves by the two-band model. **(c)** and **(d)** display the temperature dependence of carrier density and mobility, respectively.
